# Function and Regulation of IL-36 Signaling in Inflammatory Diseases and Cancer Development

**DOI:** 10.3389/fcell.2019.00317

**Published:** 2019-12-04

**Authors:** Dawn Queen, Chathumadavi Ediriweera, Liang Liu

**Affiliations:** ^1^Vagelos College of Physicians and Surgeons, Columbia University, New York, NY, United States; ^2^The Hormel Institute, University of Minnesota, Austin, MN, United States

**Keywords:** IL-36 signaling, inflammation, cytokine, psoriasis, cancer

## Abstract

The IL-36 subfamily of cytokines belongs to the IL-1 superfamily and consists of three pro-inflammatory agonists IL-36α, IL-36β, IL-36γ, and an IL-36 receptor (IL-36R) antagonist, IL-36Ra. These IL-36 cytokines function through a common receptor to modulate innate and adaptive immune responses. IL-36 cytokines are expressed as inactive precursors and require proteolytic processing to become fully active. Upon binding to IL-36R, IL-36 agonists augment the expression and production of inflammatory cytokines via activating signaling pathways. IL-36 is mainly expressed in epidermal, bronchial, and intestinal epithelial cells that form the barrier structures of the body and regulates the balance between pro-inflammatory and anti-inflammatory cytokine production at these tissue sites. Dysregulation of IL-36 signaling is a major etiological factor in the development of autoimmune and inflammatory diseases. Besides its critical role in inflammatory skin diseases such as psoriasis, emerging evidence suggests that aberrant IL-36 activities also promote inflammatory diseases in the lung, kidneys, and intestines, underscoring the potential of IL-36 as a therapeutic target for common inflammatory diseases. The role of IL-36 signaling in cancer development is also under investigation, with limited studies suggesting a potential anti-tumor effect. In this comprehensive review, we summarize current knowledge regarding the expression, activation, regulatory mechanisms, and biological functions of IL-36 signaling in immunity, inflammatory diseases, and cancer development.

## Introduction

The interleukin-1 (IL-1) cytokine family is comprised of immune-activating cytokines that regulate intercellular communication during an immune response. IL-36 is a subfamily of the IL-1 superfamily and consists of IL-36Ra, IL-36α, IL-36β, and IL-36γ (Dinarello et al., 2010; Gresnigt and van de Veerdonk, 2013). IL-36 cytokines bind to the IL-36 receptor (IL-36R) and use the IL-1 receptor accessory protein (IL-1RAcP) as a co-receptor. IL-36α, IL-36β, and IL-36γ act as IL-36R agonists, while IL-36Ra functions as a receptor antagonist that inhibits the activation of IL-36R signaling. Human IL-36 genes are located at the IL-1 locus on chromosome 2q13 and share 36–46% sequence identity with IL-1 cytokine genes (Smith et al., 2000; Gabay and Towne, 2015). IL-36 agonists induce inflammatory responses by activating nuclear factor kappa B (NF-κB) and mitogen-activated protein kinases (MAPK) via intracellular signaling cascade (Towne et al., 2004). In contrast, IL-36Ra suppresses pro-inflammatory signaling by binding to and inhibiting IL-36R activation (Towne et al., 2004; Ali et al., 2007; Kono and Rock, 2008; Sims and Smith, 2010; Afonina et al., 2015). IL-36 cytokines are mainly expressed in skin, bronchial epithelium, brain tissue, gut, and monocytes/macrophages, and play an important role in tissue homeostasis and inflammation (Smith et al., 2000; Blumberg et al., 2007; Barksby et al., 2009; Towne and Sims, 2012; van de Veerdonk and Netea, 2013; Nishida et al., 2016).

While the IL-36 signaling pathway shares key features with the IL-1 signaling pathway, disease manifestations caused by aberrant IL-36 signaling differ significantly from those due to aberrant IL-1 signaling, suggesting that these two signaling pathways affect distinct biological processes. Apart from their role in inflammatory skin diseases such as psoriasis, emerging evidence suggests that aberrant activation of IL-36 signaling also promotes inflammatory diseases affecting the lung, kidneys, and intestines, underscoring the potential of IL-36 as a marker to assist disease diagnosis and as a therapeutic target. The role of IL-36 signaling in cancer development is also under investigation, with limited studies suggesting a potential anti-tumor effect. Despite these recent progresses, our understanding of the physiological function(s), signaling mechanism(s), and molecular targets of IL-36 remains limited. In this review, we summarize current knowledge regarding the expression, activation, regulatory mechanisms, and biological functions of IL-36 signaling in immunity, inflammatory diseases, and cancer development.

## Il-36 Family and Tissue Distribution

**FIGURE 1:**
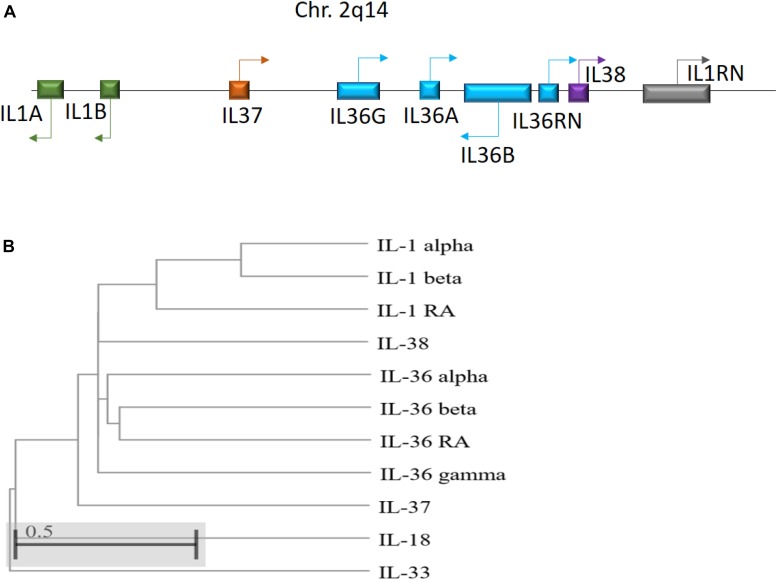
**(A)** Schematic illustration of the human IL-1 family gene cluster. Chromosomal locations and organization of the genes encoding the human IL-1 family members. Arrows indicate the direction of transcription. **(B)** Phylogenetic relationship among the 11 human IL-1 family members. The phylogenetic tree was generated based on protein sequence alignment using Cobalt tool (www.ncbi.nlm.nih.gov/tools/cobalt/re_cobalt.cgi) with Cladogram tree method and default settings.

IL-36 genes are located on human chromosome 2q13 in an IL gene cluster containing *IL1A - IL1B - IL37 - IL36G - IL36A - IL36B - IL36RN - IL38 - IL1RN*, with IL-36 encoding genes transcribed away from the centromere (Nicklin et al., 2002; Gabay and Towne, 2015; Figure 1). Between humans and mice, IL-36 subfamily members share sequence homology 54–91% of the time, with similar gene location and general organization at the IL-1 and IL-36 loci, reflecting the conserved roles of these cytokines across species (Taylor et al., 2002). Notably, IL-36 cytokines do not possess a leader sequence (Palomo et al., 2015). Instead, IL-36 cytokines share a common C-terminal three-dimensional structure with a typical β-trefoil fold consisting of 12-β-strands connected by 11 loops (Garlanda et al., 2013). IL-36α, IL-36β, and IL-36γ share 21 to 37% sequence homology with IL-1 and IL-1Ra, whereas IL-36Ra shares 54% homology with IL-1Ra (Kumar et al., 2000; Nicklin et al., 2002).

IL-36 cytokines are expressed in a variety of cell types including keratinocytes, monocytes, and dendritic cells (DCs) (Table 1). IL-36α is expressed during embryonic development and is highly abundant in epithelial cells, as well as monocytes, B cells, and T cells (Smith et al., 2000; Debets et al., 2001). In the lungs of mice, IL-36α has been observed to exert strong pro-inflammatory effects (Ramadas et al., 2012). Presence of IL-36α in these tissues, along with its regulation by epidermal growth factor (EGF), highlights its important role in skin barrier function and homeostasis (Smith et al., 2000; Li et al., 2010; Yang et al., 2010; Vigne et al., 2011; Franzke et al., 2012).

**TABLE 1:** IL-36 family cytokines.

**Cytokine**	**Tissue expression**	**Activating proteases**	**Human gene**	**Chromosome location**	**Main function**	**Disease association**	**Protective effects**
IL-36α	B lymphocytes, bone marrow, intestine, inflamed synovium, lymph nodes, monocytes, respiratory tract, skin, spleen, T lymphocytes, tonsils	Elastase Cat G	IL36A	2q12-q14.1	Pro- inflammatory	• Ulcerative colitis • Acute kidney injury • Renal interstitial fibrosis • Pulmonary fibrosis • Lung inflammation • Psoriatic arthritis • Rheumatoid arthritis	• Anti-tumor in HCC • Anti-tumor in colorectal cancer • Anti-tumor in ovarian cancer
IL-36β	B lymphocytes, bone marrow, colon, heart, inflamed synovium, lung, monocytes, neuron/glial cells, skin, T lymphocytes, testis, tonsils	Cat G	IL36B	2q14		• Epidermal hyperplasia • Cutaneous inflammation • Hyperkeratosis • Pulmonary fibrosis • Lung inflammation • Psoriatic arthritis • Rheumatoid arthritis • Ankylosing spondylitis	
IL-36γ	Bronchial epithelial cells, inflamed synovium, keratinocytes, peripheral blood lymphocytes, skin, THP-1	Proteinase 3 Elastase Cat S	IL36G	2q12-q21		• KEY biomarker in psoriasis • Ulcerative colitis • Pulmonary fibrosis • Lung inflammation • Asthma	• Acute skin wound healing • Protective against obesity • Anti-tumor in breast cancer • Anti-tumor in melanoma • Anti-tumor in colorectal cancer

IL-36β is also expressed in epithelial cells and is regulated by EGF (Franzke et al., 2012). However, in addition, IL-36β expression has been detected in murine neurons and glial cells, although neither lipopolysaccharide (LPS) nor IL-1β stimulation upregulates IL-36β expression, suggesting that it has limited activity in the brain (Berglöf et al., 2003; Wang et al., 2005). Interestingly, in human myelomonocytic cells and DCs, IL-36β expression has been linked to cellular maturation (Mutamba et al., 2012). As well, both macrophages and T cells have been found to express IL-36β (Sims and Smith, 2010; Vigne et al., 2011, 2012; Gresnigt and van de Veerdonk, 2013; Boutet et al., 2016).

IL-36γ is expressed in stimulated keratinocytes and squamous-cell epithelia of the esophagus (Kumar et al., 2000; Debets et al., 2001). IL-36γ is also constitutively expressed in DCs (Bachmann et al., 2012). Notably, LPS can effectively induce IL-36γ expression in human monocytic THP-1 cells, highlighting its role in the innate immune response (Barksby et al., 2009). Similarly, IL-36γ is expressed by peripheral blood lymphocytes when exposed to extracellular (211 At) α-particles (Turtoi et al., 2010). During chronic contact hypersensitivity and infection with herpes-simplex virus, expression of IL-36γ is upregulated and activates the pro-inflammatory NF-κB pathway (Dunn et al., 2001).

Conversely, IL-36R, the receptor for these ligands, is expressed in many different cell types with some species-specific differences between humans and mice. Human blood monocytes, myeloid dendritic cells (mDC), and monocyte-derived DCs (MO-DC) express IL-36R and respond to IL-36, whereas human T cells or neutrophils do not (Foster et al., 2014). This finding suggests T cell activation is driven indirectly, possibly through the interaction of IL-36α with MO-DC cells. Another study in human cells confirmed the expression of IL-36R in endothelial cells including dermal microvascular cells, which are known to contribute to the developed vascular networks of psoriasis (Bridgewood et al., 2017). IL-36R is also highly expressed in human M0 and M2 macrophages, but not in M1 macrophages. Consistent with this pattern, IL-36 stimulation increases production of inflammatory cytokines from M2 macrophages and directs them to a proinflammatory phenotype but has no effect on M1 macrophages (Dietrich et al., 2016). A high level of IL-36R has also been detected in human Langerhans cells (LC) and they respond strongly to IL-36β stimulation (Dietrich et al., 2016), suggesting a role for LCs in inflammatory skin phenotypes that has yet to be elucidated. Most recently, Catapano et al. (2019) demonstrated that plasmacytoid dendritic cells (pDCs) preferentially express IL-36R and binding by IL-36 potentiates Toll-like Receptor (TLR)-9 activation and IFN-α production.

In contrast to IL-36R agonists, IL-36Ra is an anti-inflammatory cytokine encoded by the IL-36RN gene that binds to and inhibits IL-36R. IL-36Ra is highly expressed in the skin, brain, tonsils, spleen, and various immune cell types such as macrophages, monocytes, B cells, and DCs (Mulero et al., 1999; Smith et al., 2000; Debets et al., 2001; Carrier et al., 2011; Lian et al., 2012; Table 1). In bone marrow-derived DCs (BMDCs), IL-36Ra acts as a selective inhibitor of IL-36α, IL-36β, and IL-36γ in a dose-dependent manner (Vigne et al., 2011). IL-36Ra can also regulate helper T cells by reducing their response in a non-classical dose-dependent manner (van de Veerdonk et al., 2012). In transgenic mouse models, IL-36γ-induced NF-κB activation is inhibited by IL-36Ra (Debets et al., 2001; Dunn et al., 2001; Towne et al., 2004; Blumberg et al., 2007).

## Il-36 Receptor Complex

In the IL-1 receptor family, three immunoglobulin domains are equally conserved within the extracellular segment. The heterodimeric IL-1R complex is formed by IL-1R and IL-1RAcP, the common accessory protein of the IL-1 family (Greenfeder et al., 1995; Korherr et al., 1997; Towne et al., 2004; Gresnigt and van de Veerdonk, 2013; Table 2). IL-36R belongs to the IL-1 receptor family and is also known as IL-1R-rp2 (Lovenberg et al., 1996). Since IL-36R shares sequence homology with IL-1R, it was hypothesized that IL-36R similarly needs support from an additional co-receptor to become fully functional (Debets et al., 2001). Indeed, IL-1RAcP has been recognized as the accessory co-receptor that triggers signal transduction upon IL-36 binding to IL-36R (Towne et al., 2004). The recruitment of IL-1RAcP to IL-36R is mediated by high affinity binding of IL-36 cytokines (α, β, γ) to the extracellular segment of IL-36R. The intracellular segment of IL-36R contains a Toll-like domain that is responsible for signal transduction (Lovenberg et al., 1996; Yi et al., 2016). Toll/IL-1 receptor (TIR) domains, which are present on each subunit, induce activation of intracellular signaling pathways and are phosphorylated by the dimerization of IL-36R:IL-1RacP (Towne et al., 2011).

**TABLE 2:** IL-36 receptor family.

**Receptor**	**Other names**	**Ligands**	**Human gene**	**Chromosome location**	**Description**	**Disease association**	**Protective effects**
IL-1RAcP	IL-1R3	Unknown, accessory chain	IL1RAP	3q28	Accessory receptor chain required for signaling in IL-1R, IL-33R, and IL-36R complexes.		
IL-36R	IL-1R6 IL-1Rrp2 IL-1RL2	IL-36α IL-36β IL-36γ IL-36Ra IL-38	IL1RL2	2q12	Ligand-binding receptor for IL-36 (α, β, and γ), forms the IL- 36R:IL-1RAcp complex to activate IL-36 signaling. It also binds with IL- 36Ra or IL-38 to inhibit recruitment of IL-1RAcp and IL-36 signaling.	• Intestinal fibrosis in IBD • Psoriatic arthritis • Rheumatoid arthritis	• Promotes acute intestinal wound healing • Deficiency is protective against murine psoriasiform dermatitis

The heterodimerization between IL-36R and IL-1RAcP (IL-36R:IL-1RAcP) is maintained by the hydrogen interactions between IL-36R and Asp150 for IL-36α, Asn148 for IL-36β, and Ala162 for IL-36γ. These bonds in turn facilitate the interaction of IL-36R with Ser185 of IL-1RAcP (Günther and Sundberg, 2014). Signal transduction is regulated by protein accumulation via disulfide bonds, N-linked glycosylation in the ectodomain of IL-36R, and trafficking of IL-36R to the cell surface. IL-36R signaling is decreased by a single nucleotide gene polymorphism, A471T for IL36R, which results in a substitution in the TIR domain that weakens its interaction with IL-1RacP (Yi et al., 2016). The agonistic characteristics of IL-36 (α, β, γ) and antagonistic characteristic of IL-36Ra are determined by the presence of a residue in the C-terminal portion within the loops β 4/5 or β 11/12 of their secondary structures. The agonistic residues, which typically consist of β-trefoil fold made of 12-β-strands connected by 11 loops, further facilitate the contact between IL-1RAcp and IL-36 (Dunn et al., 2003; Günther and Sundberg, 2014). In contrast, the Asp148 residue in the β11/12 loop of IL-36Ra acts as a steric obstacle that prevents heterodimerization between IL-1RAcP and IL-36R (Günther and Sundberg, 2014).

Upon agonist binding, IL-36R heterodimerizes with IL-1RacP to initiate signal transduction and activation through NF-κB and MAPK pathways (Carrier et al., 2011). In contrast, binding of IL-36Ra to IL-36R prevents the binding of IL-36 agonists and the recruitment of IL-1RacP, often leading to a non-signaling IL-36R homodimerization (Towne et al., 2011; Gresnigt and van de Veerdonk, 2013; Boraschi, 2017a,b). IL-36 agonist expression is positively correlated with phosphorylation of p38 MAPK and NF-κB proteins in different human and mouse cell culture models including Jurkat cells (Towne et al., 2004; Costelloe et al., 2008; Gresnigt and van de Veerdonk, 2013; Ding et al., 2018). In turn, IL-36R directly stimulates DCs and primes naive CD4+ T cells toward a Th1 response (Günther and Sundberg, 2014). The inflammatory effects in skin, synovial fibroblasts, articular chondrocytes, DCs, and T cells are associated with IL-36 cytokines and IL-36R.

## Processing, Secretion, and Regulation of Il-36 (α, β, γ) and Il-36Ra

**FIGURE 2:**
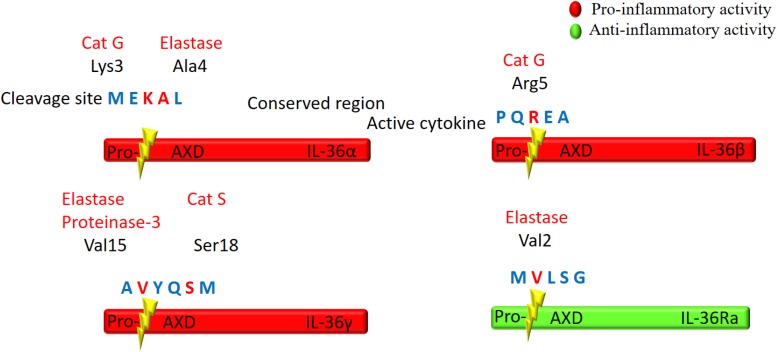
Schematic illustration of proteolytic cleavage of the IL-36 cytokines. IL-36 precursor proteins are proteolytically cleaved by the indicated enzymes to generate active mature proteins. The cleavage site is located at 9 amino acids in the N-terminal of AXD, a conserved motif. Either Cathepsin G (Cat G) or elastase can process IL-36α at Lys3 and Ala4, respectively. Cat G activates IL-36β by cleaving at residue Arg5, whereas both elastase and proteinase-3 cleave IL-36γ at Val15. Cat S can also activate IL-36γ through cleavage between Glut17 and Ser18 residues. IL-36Ra is activated by neutrophil elastase by cleavage at Val2.

IL-36 cytokines are produced as premature precursor proteins with little or no bioactivity. Pro-IL-36α, IL-36β, and IL-36γ are 1000-fold less active than their mature forms, and posttranslational processing is required for them to reach their full biological potential (Towne et al., 2011). The majority of the IL-1 family members share a caspase cutting site characterized by N-glycosylation at Asn91 and a conventional leading peptide sequence, which allows for cleavage by caspase-1 and their subsequent secretion (Barton et al., 2000; Debets et al., 2001). However, IL-36Ra and IL-36 agonists do not have characteristic cutting sites for caspase-1 or a signal peptide/pro-domain, suggesting that they utilize different secretion pathways from other IL-1 members (Mulero et al., 1999; Smith et al., 2000). Additionally, there is low homology among IL-36 cytokine protein sequences in the region of the cleavage site, suggesting multiple different enzymes may be responsible for IL-36 (α, β, γ) cytokine processing (Nicklin et al., 2002; Taylor et al., 2002; Figure 2).

Several proteases released from activated neutrophils, including neutrophil-derived cathepsin G (Cat G), elastase, and proteinase-3, can differentially process and activate IL-36 cytokines (Henry et al., 2016; Clancy et al., 2017; Table 1). IL-36 agonists can also be processed and activated by neutrophil extracellular traps (NET)-bound proteases, which possess enzymatic activity via NET-associated Cat G and elastase (Clancy et al., 2017). Both elastase and Cat G are necessary for IL-36α activation, whereas IL-36β is selectively stimulated by Cat G (Henry et al., 2016). Elastase also truncates IL-36Ra in human skin cells, leading to activation of the antagonistic form (Macleod et al., 2016). Interestingly, IL-36γ is proteolytically processed and activated by both Cat G and elastase. In human cells, however, IL-36γ is processed and activated by Cat S secreted by cells residing in barrier tissues instead of neutrophil-derived proteases. Since Cat S is readily expressed when IL-36γ is secreted, Cat S likely plays a major role in instant cleavage and activation of IL-36γ (Ainscough et al., 2017). Additionally, it has been reported that release of IL-36γ is dependent on caspase-3/7 (Lian et al., 2012).

Unlike IL-1 cytokines, IL-36 cytokines are thought to be regulated independently of the inflammasome (Towne et al., 2011). Multiple studies have shown that IL-36α and IL-36β expression in the skin is regulated by EGF signaling (Smith et al., 2000; Li et al., 2010; Yang et al., 2010; Vigne et al., 2011; Franzke et al., 2012). Additionally, Carrier et al. (2011) found that IL-36 cytokines in cultured human keratinocyte cells can stimulate self-expression in an autocrine loop. Following TNF-α, IL-17A, or IL-22 treatment in cultured human keratinocytes, IL-36α and IL-36γ levels were significantly elevated and formed a positive feedback loop with Th17 cytokines, stimulating their own expression and also the production of pro-inflammatory cytokines such as TNF-α, IL-6, and IL-8 (Carrier et al., 2011). Notably, Takaishi et al. (2018) found that immunomodulator Regnase 1 (Reg-1) could regulate and inhibit the IL-36/IL-36R auto-stimulatory loop. Indeed, Reg-1 knockout mice were found to have uncontrolled inflammation with higher IL-36α levels, suggesting a limiting role for Reg-1. Additionally, IL-36Ra antagonizes IL-36R, further regulating and inhibiting the pro-inflammatory cascade (Takaishi et al., 2018).

## Activators and Effectors of Il-36 Signaling Pathways

**FIGURE 3:**
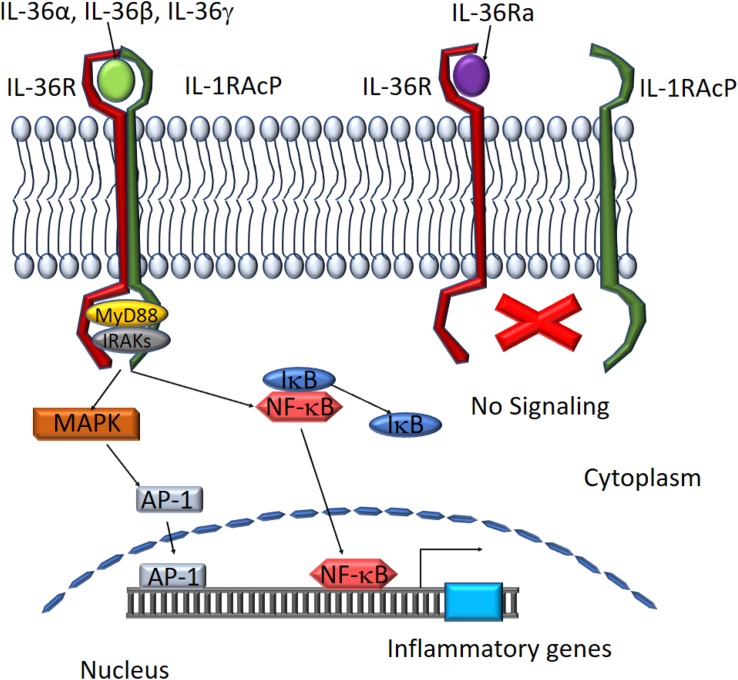
Schematic depiction of downstream mediators of the IL-36 signaling pathway. The IL-36 agonists bind to IL-36 receptor while recruiting IL-1RAcp to form a heterodimeric receptor complex. MyD88 binds to the intracellular domains of the IL-36 receptor complex and IRAKs are recruited to the complex. Activation of the MAPK pathway leads to IκB degradation and NF-κB activation. AP-1 and NF-κB translocate to the nucleus to activate target gene expression. IL-36Ra binds to IL-36R preventing the assembly of functional IL-36 receptor complexes inhibiting activation of the signaling pathway.

IL-36 agonist-mediated dimerization of IL-36R with IL-1RacP induces intracellular signaling through the engagement of cytoplasmic TIR domains of these membrane-spanning proteins. The tertiary complex formed by IL-36 cytokines and the IL-36R:IL-1RAcP receptor dimer recruits intracellular signaling molecules MyD88, IL-1R-associated kinase (IRAK), and tumor necrosis factor (TNF) receptor-associated factor 6 (TRAF6) to activate NF-κB and MAPK (Palomo et al., 2015). Activation of MAPKs, including c-Jun N-terminal kinases (JNKs) and extracellular signal–regulated kinases (ERK1/2), results in a rapid increase in the phosphorylation of IkB-α, an inhibitor of NF-κB, allowing it to dissociate and free NF-κB. This in turn leads to activation of NF-κB- and/or MAPK-dependent pro-inflammatory pathways (Towne et al., 2004; The Nguyen et al., 2012; Figure 3). Activation of MAPKs and NF-κB subsequently induces the production of other cytokines, chemokines, and anti-microbial peptides to amplify the pro-inflammatory response. IL-36Ra, on the other hand, competes with IL-36 cytokines for IL-36R binding to suppress IL-36 signaling (Günther and Sundberg, 2014; Figure 3).

IL-36 expression is highly inducible in response to multiple internal and external factors and signaling pathways. For instance, various epithelial cell types express IL-36 cytokines in response to microbial stimuli and TLR activation (Chustz et al., 2011; Huynh et al., 2016). In dendritic cells, LPS increases both IL-36α and IL-36γ expression (Vigne et al., 2011). In epithelial cells, cellular damage leads to upregulation of IL-36γ (Carrier et al., 2011; Tortola et al., 2012; Medina-Contreras et al., 2016). Similarly, skin damage activates the EGF receptor ligand and is followed by elevated IL-36α and IL-36β expression in the lesions (Franzke et al., 2012).

Following upstream activation of the IL-36 signaling pathway, IL-36 cytokines exert their pro-inflammatory effects via numerous downstream effectors that mediate the recruitment and activation of a variety of immune cells (Medina-Contreras et al., 2016; Russell et al., 2016; Aoyagi et al., 2017; Harusato et al., 2017; Kovach et al., 2017; Scheibe et al., 2017; Bassoy et al., 2018). Intradermal injection of IL-36 upregulates a host of cytokines, chemokines, and anti-microbial proteins such as beta-defensins (BD) 2 and 3, LL37, and S100A7 in an IL-36R-dependent fashion in mice. Following treatment with IL-36, human keratinocytes increased the expression of chemotactic agents such as CXCL1, CXCL8, CXCL10, CCL2, CCL3, CCL5, and CCL20 (Towne et al., 2004; Foster et al., 2014; Huynh et al., 2016; Scholz et al., 2018). In DCs, IL-36 stimulated the production of IL-10, CXCL10, TNF-α, COX-2, and granulocyte-macrophage colony-stimulating factor (GM-CSF). In human T cell/MDC allogeneic co-cultures, IFN-γ secretion is an indirect response to IL-36 stimulation (Mutamba et al., 2012).

When homeostasis is lost, these IL-36 downstream effectors have the potential to play a central role in disease. In mouse skin, for example, IL-36 stimulates production of chemotactic agents for activated leukocytes, promoting leukocyte infiltration and acanthosis of the skin (Foster et al., 2014). At the same time, excess IL-36β production can induce stromal cells to produce keratinocyte mitogens that cause keratinocyte hyperproliferation and lead to skin hyperplasia (Yang et al., 2010). As well, IL-36γ can enhance psoriatic inflammation by activating endothelial cells and promoting leukocyte recruitment in the dermal vascular compartment, while also inducing pro-inflammatory cytokines and chemokines (Li et al., 2014; Bridgewood et al., 2017). Likewise, IL-36 in psoriasis models increases expression of cathelicidin mRNA, a key player in the innate immune response (Johnston et al., 2011).

## Il-36 Functions and Tissue Homeostasis

The IL-36 interleukins are most active in barrier tissues (like the skin, lung, and intestines), suggesting that their main responsibility is to regulate the interaction of the environment and the body. Their response to microbial and extracellular ligands and subsequent production of pro-inflammatory mediators confirms this role. They may also be important in linking the innate and adaptive immune systems. Indeed, one study in mice found that among the IL-1 family of cytokines, only IL-36 could potently induce IL-2 production, T cell survival, and polarization of naive T cells (Th0 cells) toward the T helper 1 cell phenotype (Vigne et al., 2012). Under homeostatic conditions, these pro-inflammatory effects can be beneficial for fighting off infection and promoting healing and are balanced by the opposing anti-inflammatory effects of IL-36Ra.

In normal skin, IL-36 cytokines are constitutively expressed at low levels. When skin injury occurs, RNAs from damaged cells activate toll-like receptor 3 and TIR-domain-containing adapter-inducing IFN-β (TRIF), which increases the production of IL-36γ. IL-36γ then induces REG3A, which regulates keratinocyte proliferation, differentiation, and wound re-epithelialization (Lai et al., 2012; Russell et al., 2016; Jiang et al., 2017). However, when these cytokines are up-regulated and overexpressed in certain disease states, they may lose homeostatic balance, leading to a pathological pro-inflammatory milieu. In transgenic mouse skin with overexpression of IL-36α, basal keratinocytes display acanthosis and hyperkeratosis, providing evidence of the balance between skin barrier function and destruction (Blumberg et al., 2007).

Similarly, in the intestine, IL-36γ has been shown to have both pro-inflammatory effects and a potential protective role. One study suggested that IL-36γ inhibited the differentiation of regulatory T-cells, causing a loss of self-tolerance which could potentially contribute to inflammation in the intestine. In contrast, in a mouse model of colitis (intestinal), IL-36R provided protection against bacterial infection (Russell et al., 2016). Additionally, IL-36R can stimulate IL-22 production which leads to epithelial proliferation and restitution, induces secretion of antimicrobial peptides, and is protective against intestinal inflammation, indicating that IL-36 signaling is beneficial during acute intestinal wound healing (Medina-Contreras et al., 2016; Ngo et al., 2018). These studies demonstrate that the pro-inflammatory effects of IL-36 expression have the potential to be harmful, but under homeostatic conditions, low levels of pro-inflammatory signaling are in fact beneficial for intestinal immunity and wound healing.

Furthermore, in the gut microbiome, IL-36 cytokines may play a protective role in preventing obesity and metabolic syndrome. Giannoudaki et al. (2019) found that IL-36γ serum levels were increased in clinically obese patients. However, in these patients with type 2 diabetes, serum levels were negatively correlated with hemoglobin A1c (HbA1c) and fasting blood glucose (FBG) levels, suggesting that these cytokines are protective against obesity. Confirming this theory, mice with IL-36Ra deficiency showed reduced weight gain and metabolic dysfunction. The authors posited that IL-36 exerts these positive effects by promoting the growth of protective *Akkermansia muciniphila* bacteria in the gut and also enhancing colonic mucus secretion (Giannoudaki et al., 2019).

In contrast, IL-36Ra and IL-38, which can also bind IL-36R, function as negative regulators of the pro-inflammatory signaling pathway by inhibiting the dimerization of IL-36R:IL-1RAcP (Bensen et al., 2001; Kumar et al., 2002; van de Veerdonk et al., 2012). These anti-inflammatory properties have been shown *in vitro*, where addition of IL-36Ra and IL-38 to peripheral blood mononuclear cells stimulated by *Candida* demonstrated a reduction in production of T-cell cytokines IL-17 and IL-22 (van de Veerdonk et al., 2012). Thus, it is likely that homeostasis throughout different tissues is maintained by a balance of IL-36Ra and IL-36 activity.

## Dysregulation of Il-36 Signaling in Inflammatory Diseases

While normal IL-36 signaling helps maintain tissue homeostasis by promoting wound healing and tissue repair, aberrantly elevated IL-36 signaling has been associated with diverse inflammatory diseases. Psoriasis has been extensively studied and is a valuable model for understanding how dysregulated IL-36 activity contributes to chronic cutaneous inflammation involving both innate and adaptive immune responses (Towne and Sims, 2012). More recent studies have established the role of IL-36 signaling in the pathogenesis of other inflammatory diseases such as inflammatory bowel disease (IBD), acute kidney injury (AKI), and pulmonary fibrosis, as discussed below.

### Psoriasis

IL-36 interleukins have been strongly implicated in the pathogenesis of psoriasis. They are thought to exhibit their effects through activation of DCs that can promote the T helper 17 (Th17) phenotype, a key player in this disease (Tortola et al., 2012). IL-17 production by the Th17 cells may conversely up-regulate IL-36 expression, creating a feedback loop that drives inflammation and disease (Carrier et al., 2011). It has been hypothesized that this mechanism would explain the spectrum of disease, with localized activation causing Th17-dependent overexpression of IL-36 cytokines in plaque psoriasis, and systemic activation causing the more severe disease generalized pustular psoriasis (GPP) (Mahil et al., 2017).

Multiple studies suggest that IL-36γ is a potentially key biomarker for diagnosing psoriatic skin lesions (Blumberg et al., 2010; D’Erme et al., 2015; Ainscough et al., 2017). In murine-induced psoriasis models, IL-36γ activates dermal DCs and macrophages and induces the production of IL-23 and TNFα, which in turn activate T cells and drive inflammation (Clark and Kupper, 2006; Stratis et al., 2006; Wang et al., 2006; Zaba et al., 2009; Fuentes-Duculan et al., 2010; Towne and Sims, 2012; Foster et al., 2014). In skin lesions of psoriatic patients, gene expression of IL-36 has a positive correlation with Th17 cytokines, indicating a pathogenic role for IL-36 in driving Th1 and Th17 responses (Carrier et al., 2011). Furthermore, Keermann et al. used whole transcriptome analysis in plaque psoriasis patients to identify a specific pattern of gene upregulation associated with innate immunity. They found that IL-36γ and IL-36RN gene expression were significantly associated with psoriasis, exhibiting highest levels in lesional compared to non-lesional skin. On the protein level, consistent with gene expression, IL-36γ levels were markedly increased (Keermann et al., 2015b). Interestingly, Keermann et al. also found that IL-37 expression was decreased in these lesions, and IL-38 had no significant association with psoriasis. These findings together suggest that IL-36 cytokines have a pro-inflammatory role and IL-37 may have an anti-inflammatory role in this disease (Keermann et al., 2015a). Another study of psoriatic lesional and non-lesional skin used RNA sequencing and quantitative real-time PCR to analyze the differential expression of RNA splicing variants. Robust expression of disease-specific transcripts for IL-36RN and IL-36γ was observed in the psoriatic lesions, confirming their role in inflammation and disease pathogenesis (Koks et al., 2016). Lastly, Traks et al. (2019) further investigated IL-36γ gene polymorphisms in psoriasis and found three significantly associated single nucleotide polymorphisms (SNPs) and two haplotypes. Interestingly, none of the SNPs were found to directly affect the peptide sequence of IL-36γ, suggesting that they may instead affect regulatory control over the gene or the conformation of the protein (Traks et al., 2019).

IL-36α and IL-36β have also been implicated in psoriasis. In mice that overexpress IL-36α, IL-17, IL-22, and IL-23 are highly induced, sustaining a self-amplifying cytokine-expression loop (Henry et al., 2016). In addition, IL-36β may also contribute to disease as elevated levels are associated with epidermal hyperproliferation, which is consistent with clinically visible cutaneous inflammation and hyperkeratosis (Blumberg et al., 2007). Furthermore, IL-36α transgenic mice display acanthosis, hyperkeratosis, and immune cell infiltration coupled with increased expression of cytokines and chemokines (Johnston et al., 2011).

In contrast, IL-36Ra may be protective in cutaneous inflammation. Mutations in the murine IL-36RN gene have been associated with a psoriasiform dermatitis phenotype, whereas mice deficient in IL-36 or IL-36R were protected from imiquimod-induced psoriasiform dermatitis (Barton et al., 2000; Blumberg et al., 2007; Tortola et al., 2012). Similarly, in human patients, mutations in IL-36RN, which results in a misfolded IL-36Ra that disables its interaction with IL-36R, can cause the rare, life-threatening disease, GPP (Marrakchi et al., 2011; Onoufriadis et al., 2011; Farooq et al., 2013; Sugiura et al., 2013). GPP presents with sudden-onset high-grade fever, generalized rash with disseminated pustules, elevated leukocyte count, and elevated C-reactive protein serum levels (Marrakchi et al., 2011). In multiple cohorts of GPP patients, deficiency of IL-36Ra causes an increase in the severity of the lesions in the epidermis (Marrakchi et al., 2011; Kanazawa et al., 2013; Sugiura et al., 2013). *In vivo*, IL-36Ra ^–/–^ mice display more severe symptoms (Smith et al., 2000). Based on these observations, a phase 1 proof-of-concept study for seven GPP patients was conducted using BI 655130, a monoclonal antibody against the interleukin-36 receptor. Preliminary results showed significant improvement from baseline of up to a mean of 79.8% improvement by week 4 (Figure 1C). For three patients, pustules had completely cleared within 48 h of administration (Bachelez et al., 2019). This trial underscores the utility of IL-36 as a therapeutic target in psoriasis treatment.

### Inflammatory Bowel Disease (IBD)

In the United States, it is estimated that about 3 million adults suffer from IBD (Dahlhamer et al., 2016). Crohn’s disease (CD) and ulcerative colitis (UC) are two major forms of IBD associated with dysregulated innate and adaptive immune responses. In colonic mucosa, IL-36α and IL-36γ expression is elevated in UC patients (Blumberg et al., 2007). Similarly, upregulation of IL-36α and IL-36γ coupled with downregulation of IL-38 are observed in inflamed colon from CD patients (Boutet et al., 2016). In CD keratinocytes, increased IL-36γ induces TNF-α expression to sustain a self-amplifying pro-inflammatory loop with IL-17A (Frey et al., 2013). In an acute dextran sulfate sodium (DSS) induced mouse model of colitis, IL-36 deficiency reduces disease severity and decreases infiltration of innate inflammatory cells into the colonic lamina propria (Friedrich et al., 2014). In experimental colitis models and human IBD, IL-36γ expression is induced by the gut microbiota in both conditions, whereas germ-free mice do not produce IL-36γ in response to DSS-induced injury. These observations indicate a role for targeting the IL-36 signaling axis in IBD treatment. One study in mice found that IL-36R signaling played an essential role in the induction and maintenance of intestinal tissue fibrosis, a complication of chronic IBD. By targeting and preventing IL-36R activation, either genetically or by anti-IL-36R antibody-mediated blockade, they were able to significantly reduce intestinal fibrosis (Scheibe et al., 2019). These promising results suggest that further research into the effects of the IL-36 pathway during acute and chronic mucosal inflammation may facilitate future development of novel therapeutic strategies in people.

### Kidney Injury and Inflammation

The inflammatory response following acute kidney injury (AKI) precedes many pathological pathways leading to renal failure. While IL-36R expression is detected in the kidney, especially in the proximal tubules, relatively few studies have examined the role of IL-36 signaling in kidney inflammation and diseases. In a renal ischemia-reperfusion mouse model, depletion of IL-36R was shown to protect against kidney inflammation via reduction of proinflammatory cytokines such as IL-6. In unilateral ureter obstruction (UUO) mouse studies or folic acid-induced kidney injury mouse models, IL-36α mRNA and protein expression are increased significantly within 24 h of UUO and also correlated with renal dysfunction following folic acid-induced AKI (Ichii et al., 2010). In chronic glomerulonephritis mouse models, IL-36α levels correlate with the severity of the tubular damage and are indicators of renal interstitial fibrosis (Ichii et al., 2010; Chi et al., 2017). In primary cultures of renal tubular epithelial cells, IL-36α treatment increases NF-κB activity and Erk phosphorylation, providing some mechanistic insights into the effected molecular pathways (Nishikawa et al., 2018). Based on consistent upregulation of IL-36α in various mouse models of kidney diseases, including lupus nephritis, diabetic nephropathy, and traumatic kidney injury in AKI, blockage of IL-36 signaling could serve as a potential therapeutic target. IL-36α might also serve as a useful biomarker for more accurate and earlier detection of kidney injuries, even though its pathological function awaits further elucidation (Ichii et al., 2010).

### Lung Inflammation

Growing evidence suggests that aberrant IL-36R signaling contributes to the development of inflammatory lung processes. IL-36γ is highly expressed by bronchial epithelial cells in response to other cytokines, bacteria, rhinovirus infection, and smoke (Russell et al., 2016; Scheibe et al., 2017). In human bronchial epithelial cell culture, the TNF-IL-17 combination or dsRNA induces strong IL-36 (α, β, and γ) expression (Murrieta-Coxca et al., 2019). In normal human lung fibroblasts, IL-36 can then stimulate secretion of high levels of inflammatory cytokines and chemokines including IL-8, CCL2, CXCL10, G-CSF, GM-CSF, and IL-6 (Chustz et al., 2011). IL-36 can also augment the neutrophilic inflammatory response in the airways. Indeed, IL-36α and IL-36γ expression in the airway was triggered by virally stimulated TLR3 activation, leading to recruitment of Th17 cells and activation of fibroblasts, amplifying the inflammatory response (Chustz et al., 2011). Furthermore, stimulation of bronchial, endothelial, and alveolar epithelial cells with IL-36 (α, β, and γ) induces the production of inflammatory mediators and the recruitment of neutrophils and lymphocytes, activating fibrotic pathways and leading to pulmonary fibrosis (Sun et al., 2013). Similarly, asthma patients infected with rhinovirus demonstrate higher levels of IL-36γ expression in bronchial epithelial cells compared to infected cells from healthy controls. This finding suggests that IL-36 production in lung epithelial cells is stimulated by inflammatory environmental stimuli in asthma patients (Bochkov et al., 2010; Gabay and Towne, 2015). Interestingly, in a murine model of house dust mite-induced allergic inflammation, intratracheal injection of recombinant IL-36γ led to neutrophilic but not eosinophilic influx, which highlights the role of IL-36γ in controlling neutrophilic airway inflammation (Chustz et al., 2011; Ramadas et al., 2011, 2012).

### Arthritis

While psoriasis is an autoimmune disease primarily affecting the skin, psoriatic arthritis (PsA) is a major comorbidity, with around 20–30% of psoriasis patients developing PsA with skin lesions prior to joint symptoms (Busse and Liao, 2010). Compared to other forms of arthritis, PsA has the strongest correlation with IL-36 gene expression (Belasco et al., 2015). In PsA and rheumatoid arthritis (RA) patients, IL-36α is upregulated in synovium-infiltrated plasma cells which stimulate the production of IL-6 and IL-8 from synovial fibroblasts. This pathway may explain the relationship between IL-36 and induction of synovitis (Frey et al., 2013). Likewise, in RA or osteoarthritis patients, IL-36β and IL-36R expression levels are significantly elevated compared to healthy patients (Magne et al., 2006). Interestingly, experimental studies of collagen-induced arthritis (CIA), antigen-induced arthritis, TNF-induced arthritis, and K/BxN serum transfer-induced arthritis of mice show that IL-36 (α, β, and γ) and IL-36R are present, but blockade of IL-36R or IL-36R^–/–^ mice led to no improvement in arthritis. These findings suggest that the severity of experimental arthritis is independent of IL-36R, but the cytokine is likely still contributing to the inflammation (Lamacchia et al., 2013; Derer et al., 2014). Importantly, a recent study by Li et al. (2019) found that modulation of TGFβ in mice down-regulated IL-36 activity and produced a reduction in the articular cartilage degeneration and the pathological changes of OA. They found that this pathway was conserved in both murine and human systems and demonstrated that IL-36Ra can be used as a potential therapy to decrease IL-36α overactivity, arresting OA progression and leading to clinical improvement (Li et al., 2019).

In addition to affecting the joints, IL-36 may also modulate the bones themselves. In rats infected orally with *Aggregatibacter actinomycetemcomitans*, a common microbe of periodontal disease, IL-36β was upregulated in CD4+ T cells and led to osteoclastic bone resorption, an important part of the adaptive immune response (Li et al., 2010). However, high levels of IL-36β have also been implicated in pathogenic joint diseases, and polymorphisms have been noted to confer a genetic susceptibility for ankylosing spondylitis (Wu and Gu, 2007; Kim et al., 2008). A better characterization of the pathways contributing to the chronic inflammation in psoriasis, PsA, and RA will facilitate the development of new effective drugs for treating these common chronic diseases.

## Il-36 Signaling in Cancer

Despite the growing appreciation of the role of chronic inflammation in cancer development, the link between IL-36-mediated inflammation and cancer pathogenesis has not been fully elucidated. Notably, no studies to date have reported any tumor-promoting effects by IL-36, in contrast to other members of this family (Baker et al., 2019). Rather, it seems that the pro-inflammatory effects may be an important part of the anti-tumor response. Indeed, experimental studies have shown the protective effects of IL-36 in hepatocellular carcinoma (HCC), breast cancer, and melanoma, among others.

Interestingly, in HCC, a positive correlation has been observed between IL-36α expression and overall patient survival, concomitant with a negative correlation with tumor size, degree of differentiation, and tumor growth (Pan et al., 2013). In contrast, poor prognosis is associated with decreased expression of IL-36α (Pan et al., 2013), suggesting a beneficial role of IL-36α in HCC patient outcomes. IL-36α expression has also been detected in colorectal cancer tissue and IL-36α levels inversely correlate with clinicopathological parameters of colorectal cancer such as tumor size and TNM stage (Wang et al., 2014). Similarly, in epithelial ovarian cancer tissues, IL-36α was downregulated compared to controls and reduction in IL-36α expression was associated with tumor progression and poor overall prognosis (Chang et al., 2017). In contrast, IL-36α expression was shown to suppress the growth, migration, and invasion of ovarian cancer cells both *in vivo* and *in vitro* suggesting that IL-36α may also have tumor-suppressive effects.

Like IL-36α, IL-36γ may also have anti-tumor effects. The role of IL-36γ in cancer was first studied in breast cancer and melanoma, where it was shown that IL-36γ expression inversely correlated with progression of disease (Wang et al., 2015). In human colon cancer, IL-36γ was found in various types of cells within the tumor microenvironment such as immune cells, M1 macrophages, tumor cells, and vasculature cells including smooth muscle cells and high endothelial venules, which are associated with the maintenance of tertiary lymphoid structures (Weinstein et al., 2019). In particular, IL-36γ expression by macrophages is associated with markers of inflammation: fibrosis, CD4+ central memory T cell infiltration, and increased density of B cells in tertiary lymphoid structures (Weinstein et al., 2019). Therefore, IL-36γ plays a clear role in promoting a pro-inflammatory phenotype that is important in combatting colon cancer. IL-36 might also transform the tumor microenvironment and promote the differentiation of type 1 effector lymphocytes, a pivotal part of the anti-tumor immune response (Wang et al., 2015). IL-36γ expression is found to correlate with the number of tumor-infiltrating lymphocytes such as CD8+, NK, and γδT cells, and the adaptive tumor antigen-specific CD8+ T cell immune response is enhanced by IL-36γ (Wang et al., 2015), which further corroborates its anti-tumor activity.

In breast cancer cells, metastatic spread was significantly reduced when micelles loaded with IL-36γ and doxorubicin (Dox) were delivered into the lung to target lung metastases (Chen et al., 2019). This combination enhanced the type I immune response synergistically while simultaneously reducing the level of immunosuppressive myeloid-derived suppressor cells (Weinstein et al., 2017). Additionally, studies have found that when bioactive IL-36γ was injected into the tumor microenvironment, tumor progression was delayed, through the rapid recruitment of T cells and the formation of tertiary lymphoid organs (TLOs). At the sites of persistent inflammation, TLOs formed, creating a favorable microenvironment for the priming of Th0 into effector cells. The presence of TLOs was associated with improved clinical prognosis in a broad range of cancer types (Sautès-Fridman et al., 2016; Weinstein et al., 2017).

Lastly, IL-36Ra expression in the tumor microenvironment is associated with elevated intratumoral expression of PD-1, PD-L1, and CTLA4, immunosuppressive checkpoint molecules well-characterized as important inhibitors of the immune response (Weinstein et al., 2019). Since the binding affinity of IL-36γ to IL-36R is greater than that of IL-36Ra, exogenously administered IL-36γ might be able to partially mediate and downregulate expression of PD-1 and PD-L1, consequently augmenting the anti-tumor immune response and suppressing tumor growth (Weinstein et al., 2019).

## Conclusion and Future Perspectives

The IL-36 family of cytokines is a recent addition to the IL-1 superfamily and are notable for their potent pro-inflammatory effects in epithelial tissues both locally and systemically. The IL-36 signaling pathway shares key features with the IL-1 cytokine superfamily, functioning as an effective first-line immune defense mechanism. Evidence reviewed here highlights the range of functions of IL-36 cytokines in the regulation of both innate and adaptive pro-inflammatory processes in various inflammatory conditions. Recent studies on the role of different proteolytic enzymes governing IL- 36 processing have improved our understanding of the bioactivity of IL-36 cytokines. In order to maintain a healthy tissue state, IL-36 cytokines are regulated by their natural antagonist, IL-36Ra, which prevents hyper-inflammation of the corresponding tissue. When an imbalance of IL-36R and IL-36Ra occurs, inflammatory diseases might develop. A growing body of evidence from experimental models and clinical samples support the pro-inflammatory role of IL-36 in skin, lung tissue, joint synovium, and colonic mucosa tissue, which underlies the pathogenesis of psoriasis, arthritis, and IBDs. Despite the established role of inflammation in cancer development, relatively few studies have investigated the role of IL-36 signaling in cancers. Two recent studies, however, suggest a possible anti-tumor role of IL-36 in preventing cancer progression (Wang et al., 2014, 2015). Additional studies are necessary to elucidate this link. The IL-36 cytokine family also present exciting diagnostic and therapeutic potential. Targeting the IL-36 signaling axis by blocking its receptor (IL-36R) has already shown compelling anti-inflammatory effects in psoriasis, demonstrating the therapeutic potential of IL-36 in controlling inflammatory diseases. Similar preliminary studies in intestinal fibrosis and osteoarthritis also show promise. Future uses of IL-36 targeting agents in clinical trials will help define the role of IL-36 signaling in biology and disease pathogenesis, facilitating the development of new strategies for treatment of inflammatory disorders.

## Author Contributions

All authors listed have made a substantial, direct and intellectual contribution to the work, and approved it for publication.

## Conflict of Interest

The authors declare that the research was conducted in the absence of any commercial or financial relationships that could be construed as a potential conflict of interest.
